# Beneficial Effects of Childhood Selective Dorsal Rhizotomy in Adulthood

**DOI:** 10.7759/cureus.1077

**Published:** 2017-03-05

**Authors:** TS Park, Caleb Edwards, Jenny L Liu, Deanna M Walter, Matthew B Dobbs

**Affiliations:** 1 Neurological Surgery, Washington University School of Medicine, St. Louis Children's Hospital; 2 Pediatric Neurosurgery, Washington University School of Medicine, St. Louis Children's Hospital; 3 Pediatric Orthopedic Surgery, Washington University School of Medicine, St. Louis Children's Hospital

**Keywords:** adults, ambulation, quality of life, selective dorsal rhizotomy, spasticity, cerebral palsy

## Abstract

Background: Selective dorsal rhizotomy (SDR) has been used to treat children with spastic cerebral palsy (CP) for over three decades. However, little is known about the outcomes of childhood SDR in adults.

Objectives: 1) To study the effects of childhood SDR on the quality of life and ambulatory function in adult life. 2) To determine late side effects of SDR in adults.

Methods: Adults (> 17.9 years) who underwent SDR in childhood (2 - 17.9 years) between 1987 and 2013 were surveyed in 2015. Patients completed a survey, including questions on demographic information, quality of life, health, surgical outcomes, motor function, manual ability, pain, braces/orthotics, post-SDR treatment, living situation, education level, work status, and side effects of SDR.

Results: In our study population of 294 patients (18.0 - 37.4 years), patients received SDR during the ages of 2.0 - 17.9 years and were followed up 2.2 to 28.3 years after surgery. Eighty-four percent had spastic diplegia, 12% had spastic quadriplegia, and 4% had spastic triplegia. The majority (88%) of patients reported improved post-SDR quality of life and 1% considered the surgery detrimental. Most (83%) would recommend the procedure to others and 3% would not. However, patients who would not recommend SDR to others ambulated with a walker or were not ambulatory at all prior to SDR. The majority (83%) of patients improved (30%) or remained stable (53%) in ambulation. Twenty-nine percent of patients reported pain, mostly in the back and lower limbs, with a mean pain level of 4.4 ± 2.4 on the Numeric Pain Rating Scale (NPRS). Decreased sensation in small areas of the lower limbs was reported by 8% of patients, though this did not affect daily life. Scoliosis was diagnosed in 28%, with 40% of these patients pursuing treatment. Whether scoliosis was related to SDR is not clear, though scoliosis is known to occur in patients with CP and also in the general population. Only 4% of patients underwent spinal fusion.

Orthopedic surgeries were pursued by 59% of patients. The most common orthopedic surgeries were hamstring lengthenings (31%), Achilles tendon lengthenings (18%), adductor lengthenings (16%), and derotational osteotomies (16%). Twenty-four percent of all patients later underwent hip surgery and 8% had surgeries on their knees.

Conclusion: Results of this study indicate that the beneficial effects of childhood SDR extend to adulthood quality of life and ambulatory function without late side effects of surgery.

## Introduction

In the United States, selective dorsal rhizotomy (SDR) has been utilized to treat cerebral palsy (CP) spasticity over the past three decades [1-3]. SDR is the only treatment that can permanently eradicate spasticity in patients with CP. Short-term and intermediate long-term follow-up studies have shown improved motor functions after SDR in children [4-9]. However, there is little information on the long-term effects of childhood SDR on adult functional outcomes [10-11]. In the present study, we investigated long-term outcomes and quality of life for adult CP patients who underwent SDR as children.

## Materials and methods

This quality of life survey study was approved by the Institutional Review Board of Washington University School of Medicine (approval #201509071). Informed consent was obtained from patients directly or from guardians when patients were unable to provide consent. The subjects for this study were adults (age > 17.9 years) who underwent SDR in childhood (ages: 2 - 17.9 years) between 1987 and 1989 at University of Virginia Hospital or between 1990 and 2013 at St. Louis Children’s Hospital. SDR was performed through multilevel lumbosacral laminectomy in 54 patients and single level laminectomy in 240 patients [12]. Contact information was gathered from emails, mailing addresses, and phone numbers recorded in our database and medical records. Survey questions were sent out to potential participants both electronically and via letters in the mail.

The survey included questions on demographic information, quality of life, health, surgical outcomes, motor function, manual ability, pain issues, braces/orthotics, post-SDR treatment, living situation, education level, work status, and side effects of SDR. Demographic information encompassed date of birth, living situation, highest education level, and current employment status. Participants were asked if they experienced urinary incontinence, loss of sensation, and increased muscle weakness with age. Patients experiencing sensation loss were further questioned to see if the loss of sensation was complete or partial and if it caused problems with daily life. Respondents were also queried about any additional spine problems, particularly scoliosis, and if they took any steps to treat these problems.

The Diener Satisfaction with Life Scale (SWLS) was used to assess quality of life and subjective well-being by rating five statements on a scale from 1 (strongly disagree) to 7 (strongly agree). Summary scores on the Diener SWLS are interpreted as followed: (31-35) extremely satisfied with life, (26-30) very satisfied, (21-25) slightly satisfied, (20) neutral, (15-19) slightly dissatisfied, (10-14) very dissatisfied, and (5-9) extremely dissatisfied. Reliability of this scale has been reported to be greater than 0.80 [13].

Perception of one’s personal health was assessed on a five-point scale from poor to excellent as seen in Question 1 of the SF-36 health survey [14]. Opinions on SDR were assessed using yes/no questions asking patients if they felt the surgery was beneficial and if they would recommend it for other children. Yes/no questions were also used to determine if patients needed assistance with certain activities of daily life: eating, bathing/showering, using the toilet (especially if catheterization is needed to empty bladder), getting dressed, grooming/hygiene, and transferring positions. Participants were also asked other yes/no questions about whether they regularly strengthened muscles, stretched hamstrings and heel cords, or played a recreational sport. 

Motor function was assessed using the Gross Motor Function Classification Scale (GMFCS), a commonly used scale for determining gross motor function in children with CP [15]. The scale focuses primarily on functional agility related to sitting, walking, and wheeled mobility and has been validated with good correlation between levels self-reported by patients and levels determined by professionals. The five-level classification system is interpreted as followed: I - walks without limitation, II - walks with limitation, III - walks using a handheld mobility device, IV - limited self-mobility (can sit independently but is unable to stand or walk without significant support), and V - transported in manual wheelchairs with extremely limited self-mobility. 

Changes in ambulation level since surgery were ascertained by comparing preop mobility as gathered from our databases to mobility indicated on the survey via the GMFCS scale questions centered on patient’s walking ability.

Upper extremity function was assessed using the Manual Ability Classification Scale (MACS) [16]. Though this scale was originally validated in children with cerebral palsy to assess the ability to handle objects in daily life, it has also been found to have good reliability and validity in adults [17]. This five-level scale is broken down as follows: I - handles objects easily and successfully, II - handles most objects with slightly reduced quality, III - handles objects with difficulty, IV - handles limited number of objects, and V - does not handle objects.

Patients were asked to report pain experienced within the last week using the Numeric Pain Rating Scale (NPRS). The NPRS is a 0-10 scale with 0 indicating no pain and 10 being the worst pain imaginable. Participants were questioned about the location of the pain. If patients experienced back pain, they were then asked about the location of that back pain and the intensity of back pain using the NPRS. Those experiencing back pain were further queried about whether they received physician care, medications, surgery, physical therapy, or injections for their back pain. Respondents were also asked if they had constant pain in their legs and if this pain was due to muscle/joint problems or nerve pain.

## Results

The latest contact information was gathered for the 1,047 patients who met the inclusion criteria for this study from the Center for Cerebral Palsy Spasticity at St. Louis Children’s Hospital’s databases. Two hundred and thirty-seven of these patients were unable to be reached, leaving 810 potential participants to be contacted for our survey. Out of the 810 remaining patients, 294 agreed to participate and five refused. No reasons for these refusals were reported, as patients were able to anonymously deny consent to participate in this study. Additionally, due to this anonymity, the exact breakdown of individually self-reported patient responses and caregiver/guardian responses on patients’ behalf was not known. Altogether, the final 294 participant count represented 36% of potential participants who met the study criteria.

Selective dorsal rhizotomy had been performed in patients between the ages of 2.0 and 17.9 years (mean age: 7.2 ± 4.2 years). The postoperative follow-up period of this survey ranged from 2.2 and 28.3 years (mean duration: 17.2 ± 6.2 years). The ages of study participants varied from 18.0 to 37.4 years (mean age: 24.4 ± 5.4). Fifty-eight percent of these patients were male. Eighty-four percent of participants were diagnosed with spastic diplegia, 12% with spastic quadriplegia, and 4% with spastic triplegia (Table 1). 

**Table 1:** Demographic Summary of 294 Adults Who Received Selective Dorsal Rhizotomy (SDR)

Study Population	Value
Total number of patients surveyed	294
Age at surgery	2.0 – 17.9 years (mean 7.2 ± 4.2 years)
Age at follow-up survey	18.0 – 37.4 years (mean 24.4 ± 5.4 years)
Follow-up period	2.2 – 28.3 Years (mean 17.2 ± 6.2 years)
Sex	% of total patients
Male	58
Female	42
Cerebral palsy diagnosis	% of total patients
Diplegia	84
Quadriplegia	12
Triplegia	4

Sixty-six percent currently live with their parents, 14% live alone, 8% live with a roommate, and 12% either live with a significant other, caregiver, or other adult. Most patients (86%) have received a high school diploma or equivalent and 31% had an advanced degree. Forty-eight percent were still in school with 84% of this participant group reporting as full-time students. A nearly equivalent percentage of participants (45%) were employed at the time of responding with 40% working full-time and 60% working part-time (Table 2).

**Table 2:** Summary of Living, Education, and Employment

Living situation	% of total patients
With parents	66
Alone	14
With roommate	8
With significant other	6
With caregiver	3
Other	3
Education	% of total patients
Still in school	48
High school diploma	51
Advanced degree (beyond high school)	31
Employment	% of total patients
Employed	45
	% of employed patients
Full-time	40
Part-time	60

Ninety-five percent rated their health as good or better, 4% as fair, and 1% as poor (Table 3). Respondents typically gave responses in accordance with the higher end of the slightly satisfied bracket of the Diener Satisfaction with Life Scale (SWLS). The mean overall SWLS score was 25 ± 8. Seventy-seven percent of participants scored greater than neutral (score > 20), indicating that a majority of respondents were at least slightly satisfied with life. With respect to individual questions in the SWLS, participants responded with greatest agreement to the statement “I am satisfied with my life” and with least agreement to the statement “If I could live my life over, I would change almost nothing”. 

**Table 3:** Summary of Perceptions of SDR and Health SDR: selective dorsal rhizotomy

How did SDR affect your Quality of Life?	% of total patients
Increased	88
Decreased	1
No change	1
Not sure	10
Would you recommend SDR to others?	% of total patients
Yes	83
No	3
Not sure	14
Perception of health	% of total patients
Excellent	24
Very good	39
Good	32
Fair	4
Poor	1

The majority of participants in our study reported to be independent with activities of daily life. Out of all individual daily activities, patients had the most issues with grooming and hygiene (35% requiring assistance). Patients also reported physical and recreational daily activities. Sixty percent strengthened muscles at least once a week, 47% regularly stretched hamstrings, and 38% regularly stretched heel cords. Twenty-one percent of participants engaged in recreational sporting activities as well (Table 4).

**Table 4:** Summary of Patient Mobility and Activities after SDR

Gross Motor Function Classification Scale (GMFCS)	% of total patients
I	29.5
II	28
III	29.5
IV	8
V	5
Manual Ability Classification Scale (MACS)	% of total patients
I	71
II	20
III	7
IV	1
V	1
Activities of Daily Living	% of total patients
Eating	88
Bathing	68
Toileting	79
Dressing	70
Hygiene	65
Transfers	81
Activities of Daily Living	% of total patients
Regularly strengthen muscles at least once a week	60
Regularly stretch hamstrings	47
Regularly stretch heel cords	38
Play recreational sports	21

At the time of the survey, most participants (87%) were classified as Gross Motor Function Classification Scale (GMFCS) Levels I, II, and III with only 13% of patients classified as GMFCS Levels IV and V. Ninety-one percent reported to handle most objects independently and were at either MACS Level of I or II (Table 4). In total, 37% of all patients currently use orthotics, and of these patients, 61% have changed their orthotic use over the years (Table 5).

**Table 5:** Summary of Brace/Orthotics Use

Lower Limb Orthotic Use	% of total patients
Currently using orthotics	37
	% of patients using orthotics
Changed orthotics type	61

Thirty percent of participants have improved their overall level of ambulation since surgery, while 17% saw their ambulation worsen over time. The remaining 53% of respondents reported ambulation in a manner similar to their ambulation level prior to surgery (Table 6). Preoperatively, 44% of participants ambulated independently and 35% walked with the assistance of walkers (35%) (Table 7). At the survey follow-up, more patients walked independently (48%) or with crutches/canes (17%, as compared to 7% preoperatively) and less patients used walkers (22%). A small portion of patients (13%) reported that they were wheelchair-bound or had difficulty moving generally during follow-up (Table 8). Forty-one percent of respondents indicated that they were able to run.

**Table 6:** Summary of Ambulation Changes SDR: selective dorsal rhizotomy

Ambulation at the Time of Survey Compared to Pre-SDR Ambulation	% of total patients
Improved level of ambulation	30
Same level of ambulation	53
Worsened level of ambulation	17

**Table 7:** Summary of Pre-SDR Ambulation

Parameter	% of total patients
Independent ambulation in all environments	27
Independent ambulation in protected environments	17
Ambulation with crutches/canes, all environments	2
Ambulation with crutches/canes, protected environments	5
Ambulation with walker, all environments	16
Ambulation with walker, protected environments	19
Crawling, reciprocating arms and legs	6
Some method of independent locomotion	7
No independent mobility	1

**Table 8:** Summary of Post-SDR Ambulation SDR: selective dorsal rhizotomy

Parameter	% of total patients
Walk on own without walking aids wherever and can use stairs without handrails	31
Walk on own without walking aids but with difficulty on uneven surfaces, crowds, and uses handrails for stairs	17
Walk on own with crutches/canes but with difficulty on uneven surfaces, crowds, and uses handrails for stairs	7
Walk on own with walker but with difficulty on uneven surfaces, crowds, and uses handrails for stairs	4
Walks/stand only with crutches/canes with difficulty on stairs, uneven surfaces; may use wheelchair for long distances	10
Walks/stand only with walker with difficulty on stairs, uneven surfaces; may use wheelchair for long distances	18
Cannot stand/walk without significant support, uses wheelchair at home, school, community	8
Has difficulty with movement and needs to be lifted by another person to move	5

Eighty-eight percent felt that the SDR procedure was beneficial to them, while 1% considered SDR to have been detrimental to their quality of life. Overall, 83% would recommend SDR to other children with CP, 3% would not recommend the procedure, and 14% were unsure (Table 3). The primary reasons for being unsure were: inability to recall how much impact the procedure had on patients during childhood and a concern for lack of commitment on behalf of patients and families to the strenuous physical therapy following surgery. 

For the nine patients who would not recommend SDR, five were diagnosed with spastic diplegia and four with spastic quadriplegia. Five patients could ambulate using a walker prior to surgery, while the other four could utilize some method of locomotion to get around. At the survey follow-up, four of these participants reported having difficulty with movement and needing to be lifted to move while three participants could not stand or walk without significant support, requiring a wheelchair to get around. One patient was able to ambulate with a walker and another ambulated using a cane. These patients had a wide range of SWLS scores from 5 to 29 (mean score: 17 ± 10), although three patients declined to respond to the Diener Satisfaction with Life scale. 

Bone problems in the spine, primarily scoliosis, were seen in 28% of all participants with 40% of these patients pursuing some form of treatment. Only 4% of all participants underwent fusion surgery for spinal problems. Follow-up orthopedic surgeries were received by 59% of all patients. The most common orthopedic surgeries were hamstring lengthenings (31%), Achilles tendon lengthenings (18%), adductor lengthenings (16%), and derotational osteotomies (16%). Twenty-four percent of all patients also underwent hip surgery and 8% received surgeries on their knees (Table 9).

**Table 9:** Summary of Conditions and Post-SDR Medical Interventions SDR: selective dorsal rhizotomy

Parameter	% of total patients
Diagnosis of Scoliosis and Other Back Issues	28
	% of scoliosis patients
Back issue intervention for scoliosis	40
	% of total patients
Spine fusion surgery	4
Orthopedic surgery	59
Hip surgery	24
Knee surgery	8
Tendon lengthening surgery	50
Hamstrings	31
Achilles tendon	18
Adductors	16
Calf muscles	7
Derotational osteotomy	16
Baclofen pump implanted post-SDR	3
Currently implanted	2
Currently use oral spasticity medication	18
Oral Baclofen	12

Three percent of participants had baclofen pumps inserted after SDR and 2% (three quadriplegia patients and two with diplegia) still had baclofen pump implants at the time of the survey. Medication-based anti-spasticity treatments were being taken by 18% of patients (74% diagnosed with diplegia, 22% quadriplegia, and 4% triplegia) with 12% of the total study population taking oral baclofen (Table 9).

Twenty-nine percent of patients reported pain within the past week, mostly located in the back and lower limbs. The mean pain level experienced by patients was 4.4 ± 2.4 and mean back pain score was 4.9 ± 2.6 on the Numeric Pain Rating Scale of 0 - 10 with 0 representing no pain and 10 representing worst pain imaginable. Most patients did not pursue further interventions for back pain, though medications and physical therapy were utilized by 39% and 34% of individuals reporting back pain, respectively. Constant leg pain was reported by 11% of participants. The overwhelming majority (87%) of these patients indicated that this pain was due to muscle and joint problems. Thirteen percent of respondents experiencing leg pain stated that their leg pain was diagnosed as nerve pain (Table 10).

**Table 10:** Summary of post-SDR pain, bladder function, sensory changes and muscle weakness with age

Parameter	% of total patients
Patients experiencing pain	29
	Numerical Rating Scale ( 0–10)
Average pain score	4.4 ± 2.4
Where is the pain located?	% of patients with pain
Back	70
Upper limb	8
Lower limb	46
Head	12
Other	24
	% of total patients
Constant leg pain	11
Cause of pain?	% of leg pain patients
Muscle and joint problem	87
Nerve pain	13
	% of total patients
Urinary incontinence	11
Requiring catheterization	0
Decreased sensation in lower limbs	8
Muscles weakening with age	30

Urinary incontinence was seen in 11% of respondents. None of these patients used a catheter to empty their bladder, suggesting that incontinence was unrelated to the selective dorsal rhizotomy. Decreased sensation in the lower limbs was reported by 8% of patients; 29% of these patients responded after the initial survey with more details on the sensory changes. None had complete loss of sensation in their legs with most only having numbness in parts of their legs. This did not cause any problems in their daily life. Thirty percent of respondents indicated that their muscles were weakening with age (Table 10).

## Discussion

Various treatments are currently employed for spastic cerebral palsy (CP) in childhood. However, there are no long-term prospective studies in the CP literature examining the effects of selective dorsal rhizotomy alongside a variety of treatments, including orthopedic surgery, intrathecal baclofen treatment, Botox injection, antispasticity medication, and physical therapy. Lack of sufficient research on long-term outcomes may be attributed to the low prevalence of patients diagnosed with CP in the United States, which is about 1 in 323, resulting in the designation of low priority grant funding for costly long-term CP research [18]. It is unlikely that CP research funding will improve in the near future, and thus, other research options should be sought out. An alternative option to research long-term CP treatment outcomes is to conduct a survey questionnaire study asking former patients to report quality of life and functional measures. Although a survey questionnaire study has limitations as a research tool, it can produce meaningful patient information on the effects of a previous treatment that was administered years or decades earlier [11].

Strengths of our study include the examination of a large patient cohort and of a long-term follow-up period reaching adulthood for participants who received SDR as children. Our study’s 294 adult participant cohort constitutes the largest study population among all long-term SDR studies reported to date. Additionally, the follow-up period extends up to 28 years, including a cohort of 96 patients who fall in the 20 - 28-year follow-up category. Only one previous study has examined a similar long-term follow-up period in a large SDR patient population [11].

The limitations of our study are twofold. First, out of the 810 potential participants who met study criteria, 294 adults (36%) participated. Given that our study utilized a long follow-up period that involved contacting patients many years after their initial surgery, the 36% participation rate in this study was excellent. However, the outcomes of non-participants are still unknown. Second, data was collected from subjective patient self-report through an online survey questionnaire. These responses reflect the perceptions of those who completed the questionnaire and may present response bias in comparison to more objective measurement tools, such as neurological examination or personal interviews. In addition, due to the anonymity of responses, the exact questionnaire completion breakdown of patients to caregivers and guardians, on behalf of patients, is not known.

At our center, the primary goal of SDR is to improve patients’ ambulation. Therefore, our data collection was focused on ambulation status before and after SDR. It is significant that 30% of our patients reported improved ambulation since surgery and that 53% reported similar ambulation as before surgery (Table 6). It should also be noted that our ambulation outcomes did not assess qualitative changes of ambulation. For example, stride, cadence, balance, and overall gait could have improved after surgery in the absence of concurrent ambulation changes.

The remaining 17% experienced worse ambulation since surgery; however, none of the patients in this group ambulated independently or with crutches prior to surgery. This result is consistent with our clinical observations. Patients who ambulate independently or with crutches before and after SDR maintain a high level of ambulation as they age. By contrast, patients who preoperatively ambulate with walkers in only protected environments can lose the ability to ambulate as they age. The decline is caused by increased body weight with growth in the absence of concurrent increased strength.

CP spasticity also leads to “early aging” that is typically associated with decreased strength, endurance, and muscle/joint pain [19]. In our clinical experience, early aging manifests in late childhood and early adulthood. If spasticity goes untreated, patients continue to deteriorate and eventually lose the ability to walk after 50 years of age. We have not seen any patient whose signs of spastic CP abate spontaneously as they age. Thus, improved or stable ambulation levels in 83% of our patients were causally related to reduction of spasticity with SDR. Without SDR intervention, many of the patients in our study could have experienced deteriorated ambulation or even have required wheelchairs to get around. Overall, our data suggests that early SDR can prevent premature aging and improve ambulation in adulthood.

We observed 1% of our study population reporting decreased quality of life after SDR and 10% feeling unsure about how SDR impacted their quality of life (Table 3). The reasons for being unsure were that patients were too young at the time of surgery to know the effects of SDR and children or parents were not committed to the assigned physical therapy protocol following surgery. It should be noted that during the early years of our SDR practice, we were unable to predict detailed patient outcomes due to the lack of available SDR literature. Additionally, we were unfamiliar with the early aging phenomenon caused by untreated spasticity. Hence, these earlier patients received only limited information on the early aging phenomenon and probable outcomes of SDR. If patients and parents had been better informed prior to surgery, as we currently are, their reported perception of SDR could have been markedly different. Therefore, we strongly emphasize the importance of informing current and future patients of the intense physical therapy regimen required following surgery and the early aging phenomenon when suggesting SDR as a treatment option.

Though 83% of participants stated they would recommend SDR, 3% (nine patients) would not recommend the procedure (Table 3). For the nine patients who would not recommend SDR, five ambulated with a walker, and four were non-ambulatory prior to surgery. At the time of the survey, seven were non-ambulatory and required wheelchairs. One patient ambulated with a walker and another ambulated with a cane.

It is important to note that none of the nine non-recommending SDR patients were independently ambulating following SDR. This finding is not surprising. Patients who can ambulate independently after surgery would feel more positively about the surgery. However, the finding brings up a key point in that the goals of surgery and expected outcomes must be discussed in detail with patients and parents prior to SDR surgery. If it is predicted that a child is expected to walk with a walker after surgery in only protected environments, patients and parents should be informed that worsening ambulation is probable with future growth. If surgery is performed for the primary goal of comfort and pain reduction, patients and parents should understand that they may be disappointed with the functional mobility outcomes years later. 

In our patients, 29% reported experiencing pain, mostly in the back and the lower extremities. Pain was not intense in most cases. The frequency of pain was not assessed. In a similar study of adolescents and adults who underwent SDR in childhood, chronic pain affected 51% of 88 participants [11]. Other studies reported pain in 67% of 93 adults with CP, with pain often resulting from failed orthopedic surgeries [20]. In the total U.S. adult population, 50% to 80% adults experience at least one episode of back pain during their lifetime [21]. Therefore, multiple other factors could be the cause of pain in our study’s CP patients. We do not know the effect of SDR on the rate of pain in our patients. Nevertheless, the fact that a portion of CP patients will develop back and leg pain in adulthood should be a factor when considering any surgical treatment.

Though urinary incontinence was reported in 11% of our study population, previous studies have reported higher rates (33%) of incontinence in the general CP population [22]. In addition, none of the patients in our study required catheterization to empty their bladder, indicating no causal relations with SDR. CP is associated with uninhibited neurogenic bladder, whereby normal inhibitory control of detrusor function by the central nervous system is impaired or underdeveloped, resulting in urgency or enuresis [23]. If the urinary incontinence is due to SDR, perineal sensation would be absent. However, all patients exhibited intact perineal sensation, allowing us to rule out SDR as a cause of the incontinence. In addition, a tethered spinal cord is often raised as a cause of urinary incontinence. If a spine MRI in the prone position delineates ample CSF space dorsal to the surgery site, then the tethered cord is ruled out. We have not encountered a tethered spinal cord in the over 3,500 patients that have come to our center.

Though 28% of patients reported a scoliosis diagnosis at the time of the survey follow-up, there is not enough evidence in the SDR literature to suggest that scoliosis resulted from surgical intervention [24]. Moreover, the suggested incidence of scoliosis in all CP patients has been reported as 29%, which is similar to the incidence observed in our study [25].

Eight percent of our patients reported diminished sensation in small areas of the lower extremities, though the sensory changes did not affect daily living. Thirty percent reported weakening muscles with age. However, the weakness is most likely due to muscle underuse rather than SDR. In a previous study, we reported no change in strength two years after SDR, suggesting that weakness could not have been a result of nerve damage from SDR [6]. To date, there is no indication that SDR causes late motor weakness. In our survey, 60% of patients strengthened muscles at least once a week, 47% regularly stretched hamstrings, and 38% regularly stretched heel cords (Table 4). This is a positive result, but ideally, all patients should strengthen and stretch muscles daily in order to prevent muscle weakness as they age.

## Conclusions

Results of this study indicate that the beneficial effects of childhood SDR extend to adulthood quality of life and ambulatory function without late side effects of surgery.
